# Tracheoesophageal voice prosthesis management in laryngectomy patients during the COVID-19 pandemic

**DOI:** 10.1186/s40463-020-00456-z

**Published:** 2020-08-10

**Authors:** David P. Goldstein, Gilbert Ralph, John R. de Almeida, Ashok R. Jethwa, Jonathan Irish, Douglas B. Chepeha, Dale Brown, Patrick Gullane, John Waldron, Elana Aziza, Lisa Durkin

**Affiliations:** 1 Department of Otolaryngology-Head and Neck Surgery, University Health Network, University of Toronto, Toronto, ON Canada; 2 Department of Radiation Oncology, Princess Margaret Cancer Centre, University of Toronto, Toronto, Canada; 3 Speech pathology, University Helth Network, University of Toronto, Toronto, ON Canada

**Keywords:** Laryngectomy, Tracheoesophageal puncture, Voice prosthesis, COVID-19, Complications

## Abstract

With the COVID-19 pandemic, there has been significant changes and challenges in the management of oncology patients. One of the major strategies to reduce transmission of the virus between patients and healthcare workers is deferral of follow-up visits. However, deferral may not be possible in total laryngectomy patients. Urgent procedures may be necessary to prevent complications related to ill-fitting tracheoesophageal puncture (TEP) voice prostheses, such as aspiration or loss of voicing. In this paper, we describe the Princess Margaret Cancer Center’s approach to managing this unique patient population.

## Introduction

With the outbreak of the Coronavirus (COVID-19) pandemic a state of emergency was declared in Ontario on March 17, 2020 with directives from the Ministry of Health and Long-term Care to restrict all but essential ambulatory care services [1]. Since then, health care systems and teams have had to significantly adjust how they care for oncology patients in order to reduce the risk of virus transmission [2]. Changes include virtual consults, screening for COVID-19 prior to care, wearing personal protective equipment (PPE) and deferral of visits and treatments [3].

Since coronavirus is transmitted through respiratory droplets or aerosolization of virus from the upper aerodigestive tract (UADT), aerosol generating procedures (AGPs), such as those frequently performed in Otolaryngology-Head and Neck Surgery, can put health care workers at significant risk of transmission of the virus when performed in COVID-19 positive patients [4–6].

Laryngectomy patients with tracheoesophageal puncture (TEP) who use a voice prosthesis for vocal restoration require frequent specialized on-going care in addition to their oncology follow-up. Voice prostheses will eventually fail, resulting in complications, and need for replacement. The most common complication is leakage, through or around, the voice prosthesis resulting in aspiration of liquids while drinking. The life span of the device is variable between patients (median device life of 61 days) and can vary within the same patient over time [7]. Many patients using TEP voice prostheses may be able to tolerate small amounts of aspiration on a temporary basis without developing pneumonia, while a minority can experience serious negative health consequences from aspiration such as hospitalization for pneumonia or respiratory compromise [8]. Another complication of TEP voice prosthesis use is dislodgement which can result in aspiration of the voice prosthesis, aspiration of solids and/or liquids, or closure of the TEP tract requiring a future secondary procedure to recreate the fistula. Any or all of the above complications may result in patients requiring emergency department services and/or hospital admission for treatment.

In the context of the current pandemic, it is important to develop clear processes to support patients at risk for complications associated with TEP voice prosthesis use in order to guard their safety and reduce potential additional health system burden. Given the manipulation of the UADT with TEP voice prosthesis change, there is the possibility for aerosolization of virus particles [5]. In this context, TEP voice prosthesis change should be carefully considered and undertaken after all other reasonable strategies for temporary management have been employed [9, 10]. There have been several recommendations published by institutions and societies on the management of total laryngectomy patients during the COVID-19 pandemic [9, 10]. Described below is the process for inter-professional care and management of laryngectomy patients with TEP complications at the Princess Margaret Cancer Centre during the COVID-19 pandemic.

## Princess Margaret Cancer Centre approach to managing patients with TEP complications

**Fig. 1:**
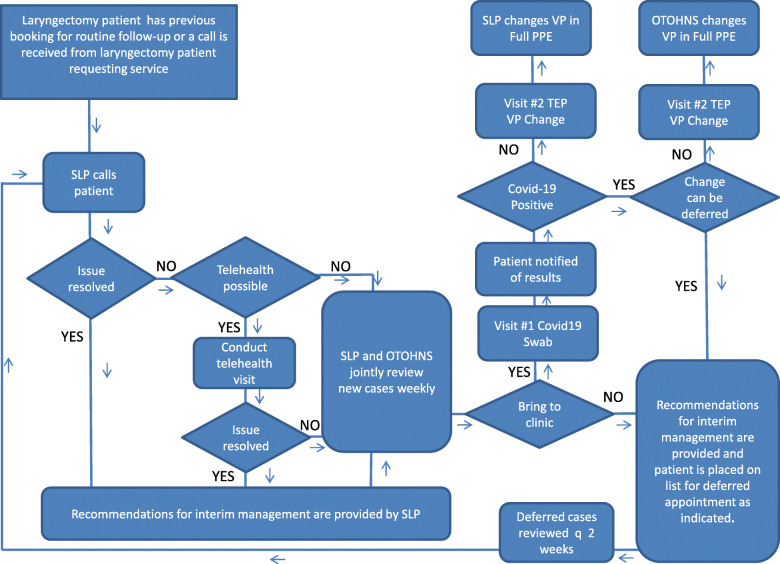
Algorithm for managing TEP complications where COVID swab screening is available

The processes described below are represented in Fig. 1. Laryngectomy patients who are booked for routine ambulatory care follow-up or patients who call to request an appointment are screened by the speech language pathologist (SLP) via an initial telephone call. The SLP determines the nature of request for service (i.e. education, assistance in ordering supplies, requests for speech therapy to improve communication function or requests to change a voice prosthesis which is leaking). Once the issue is identified, the SLP works with the patient and their family by phone to temporarily resolve the issue. For patients with a leaking voice prosthesis a variety of strategies for self-management are discussed with the patient and family. Strategies that have the highest likelihood to successfully eliminate or ameliorate aspiration with the least complexity and effort on the patient’s part are coached first. Such strategies might include cleaning the prosthesis to ensure the valve is fully closed; intentionally consuming a bite of solid food before taking a sip of liquid; use of commercially available plugs; adaptation of voice prosthesis insertion stick to serve as a plug; holding a cotton tip applicator at the lumen of the prosthesis to help absorb drops of liquid or use of thickening powders to thicken liquids that are consumed. Recommendations provided are customized to suit each patient’s unique situation. Coaching and support for implementation of the recommendations are provided by the SLP.

If the initial phone follow-up does not result in resolution of the issue, the SLP will arrange a secure video-based Ontario Telehealth Network visit, if possible. This visit has the added advantage to allow for the voice prosthesis to be viewed and for face-to-face problem solving. All requests for out-patient SLP service during the pandemic, particularly those not resolved by virtual appointments, are reviewed weekly by SLP and an assigned head and neck surgeon. In addition to the problem or concern with the TEP, other factors such as patient’s age, past medical history (e.g. comorbidities including other respiratory conditions, immunosuppression, previous history of aspiration pneumonia and severity), current health condition with particular attention to patient report of stable or deteriorating condition and signs of aspiration pneumonia (fever or discoloration of tracheal secretions) are taken into account to determine whether an in-person appointment is required. These factors not only help determine the risk of a major complication from aspiration but also the risk of morbidity or mortality should the patient be exposed to COVID-19.

If the recommendation either from the phone call or the video-based visit is to defer an in-person visit, the patient’s name is added to a deferred appointment list to be arranged when ambulatory care restrictions are lessened. Where needed, deferred patients will also be contacted 2 weeks later to continue to receive support and re-evaluation.

Patients who are negative by COVID-19 clinical screening who require an in-person visit will undergo a COVID-19 screening swab 24 to 48 h prior to their prosthesis change. Given that tracheal swabs are not routinely performed, screening of laryngectomy patients is usually performed in clinic by the surgeon or their trained delegate. Laryngectomy patients will undergo swabs of the trachea in addition to the oropharynx and nasal cavity/nasopharynx [9]. This can be done using either separate swabs or a single swab (start with the trachea, followed by oropharynx and lastly the nasopharynx). Patients will then be sent home to self-isolate until their return appointment within 48 h once COVID-19 negative status is confirmed. At this visit the patient’s voice prosthesis will be changed by SLP using full PPE (level 2 gown, gloves, N95 Mask and a face shield). Even if patients are negative on their screening test, full PPE precautions are undertaken when performing voice prosthesis change as sensitivity of nasal swab and/or tracheal screening tests in asymptomatic patients may vary depending upon where patients are in their infection course should they be infected and the fact that an AGP is being performed [11]. While topicalization with local anesthetic spray is frequently used in the non-COVID setting, currently we have moved away from using lidocaine spray and if needed either topicalize the airway with a lidocaine soaked pledget or inject lidocaine around the TEP site. Fortunately, most patients are well-known to the head and neck SLP team who will thus know the extent to which they require topicalization and how easy the TEP change will be. After completion of the visit, the examination/procedure room will be cleaned based on the hospitals infection, prevention and control (IPAC) guidelines.

Patients who screen positive for COVID-19 will be advised of their status and instructed to continue to self-isolate. The IPAC team will be informed of the result and hospital protocols related to the COVID-19 positive patient should be followed. The SLP and surgical team will review the case in further detail to determine if prosthesis change can be deferred without significant medical compromise. Decisions regarding re-evaluation and re-swabbing will be made in conjunction with IPAC following hospital guidelines. Patients who are deemed to critically require immediate prosthesis change will be discussed with IPAC and undergo prosthesis change by a member of the surgical team in full PPE, with preference to use a negative pressure room and a powered air purifying respirator (PAPR) where available. A terminal clean of the room will be required.

## Discussion

**Fig. 2:**
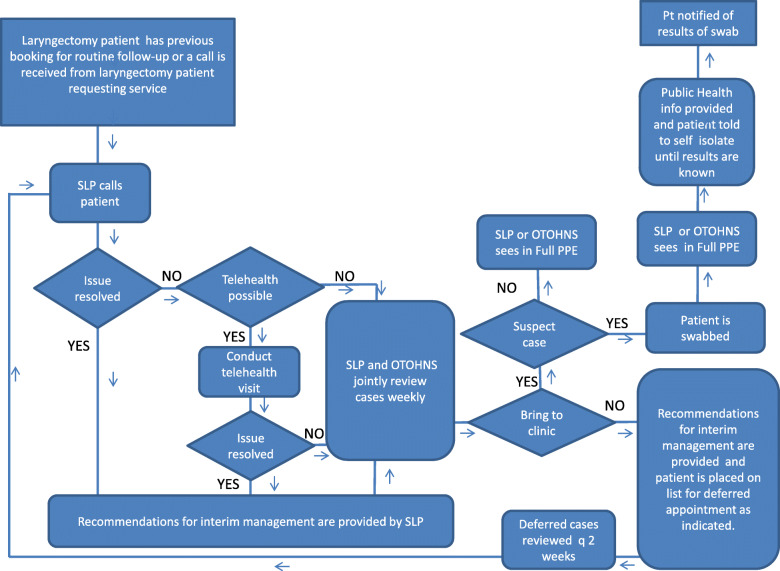
Algorithm for managing TEP complications where COVIS swab screening is NOT available

The protocol that was developed at the Princess Margaret Cancer Centre has undergone a number of iterations based on availability of PPE as well as accessibility to COVID-19 testing. The algorithm outlined in Fig. 1 is the most up to date and is similar to that described by Hennessey et al. For centers without the availability of COVID-19 screening tests, Fig. 2 presents our initial algorithm prior to the implementation of screening tests.

As noted by Hennessey et al, and others, deferral where possible is the best management option for reducing the risk of transmission of COVID-19 [9, 10, 12]. However, deferral may not always be feasible for patients at risk for potential serious complications of prosthesis failure, such as frail, elderly patients or patients with multiple co-morbidities. These patients require face-to-face evaluation and management. Prior to being brought to clinic, patients undergo a verbal screening for COVID-19 symptoms. Difficulty arises from the fact that the symptoms of aspiration (dyspnea, cough and fever) can be similar to those of COVID-19. For these patients, laboratory screening tests are critical. As screening tests become more readily available, pre-screening should be possible. However, in the event a patient requires intervention before results from a laboratory screening test are available; the patient should be managed via the COVID-19 positive arm of Fig. 1.

While the above screening algorithm works well for patients that live within close proximity to the center, some head and neck patients reside at a distance and thus having two visits in a short time period is not often feasible. This highlights the importance of virtual consults, particularly using telehealth platforms that allow person to person consultation to troubleshoot problems without having to come to hospital. For those that do require an in-person visit, testing at a local screening center may be a possibility prior to the appointment depending on availability. If the latter is not available, patients may require two visits or less favorably the patient may be seen without screening but precautions taken assuming the patient is COVID-19 positive.

Screening for COVID-19 in the laryngectomy patient population also represents a unique situation given that the UADT is discontinuous. While there is no data to suggest that tracheal and nasopharyngeal swabs will be discordant in laryngectomy patients, there are reports in the general population of COVID-19 positive patients demonstrating that nasopharyngeal swabs and tracheal aspirates can be discordant [13]. Given this uncertainty, we recommend swabbing both sites similar to the technique by Hennessey et al. [9]

One way to potentially reduce transmission to health care workers and reduce community spread in the laryngectomy patient is to cover the tracheostoma with a heat moisture exchanger (HME), and as noted by Hennessey et al*,* preferably with an integrated viral/bacterial hydroscopic filter, and a physical barrier over the stoma prior to their arrival to the clinic. Further discussion of available devices as they relate to COVID-19 is presented by Hennessey *et al* [9] and Parrinello et al. [12] In addition, HME devices reduce crusting and potential trips to hospitals to address obstruction related to such.

For the dislodged prosthesis when there is concern for aspiration of the prosthesis, patients can be seen at their local emergency department to undergo chest imaging (Chest x-ray or Chest Computed Tomography) and can undergo COVID-19 testing there, if available. Unless patients are seen at a specialized head and neck cancer center, it is unlikely that SLP services will be available to manage a dislodged prosthesis. In such cases, patients can be managed by a physician with insertion of a catheter (Foley or red rubber catheter) through the TEP. Hennessey et al suggest that patients with a dislodged (non-aspirated) voice prosthesis may also be conservatively managed at home by self-insertion of a red rubber catheter or dilator into the TEP tract, for patients familiar with this procedure until TEP replacement becomes a viable option [9]. Whether the catheter is placed by a local physician or by the patient, the patient should contact their primary SLP for phone evaluation and further management as indicated.

Catheter placement can also be considered to temporarily manage leakage through or around the voice prosthesis. This approach involves removal of the prosthesis followed by insertion of the catheter. While the catheter placement might temporarily manage leakage, it does not eliminate the need for airway manipulation as the leaking voice prosthesis needs to be removed and the catheter needs to be inserted through the TEP. Insertion of the catheter may eliminate leakage for many patients, however, some patients may continue to leak around a catheter, thereby not addressing the underlying problem. In addition, if the catheter becomes dislodged and the patient is not able to reinsert it themselves, they will require a face-to-face visit for the catheter to be reinserted, resulting in additional health care burden and potential exposure for the patient. Additionally, for patients who use a device such as a laryngectomy tube or stoma button, the presence of the catheter may make stoma care and use of an HME more difficult, which in turn could increase the risk for mucous plugging and need for further health care intervention. For these reasons, we do not routinely recommend removal of a leaking voice prosthesis and placement of a catheter as first line management; direct replacement of a voice prosthesis may have a greater likelihood to completely resolve the issue without significantly more airway manipulation.

One particular challenge is the COVID-19 positive patient that is having significant aspiration and is at risk for pneumonia from both aspiration as well as the coronavirus. Careful consideration by the SLP and surgical team is needed with regard to bringing these patients in and changing their prosthesis. We have made the provision that if there is significant concern that the TEP complication is life threatening in a COVID-19 positive patient, the patient will be brought in for management under full protective guidelines. If a voice prosthesis change is required, it is to be performed by the staff head and neck surgeon and/or the most experienced SLP with as few people as possible to be involved in the change. COVID-19 positive patients deemed necessary to undergo immediate prosthesis change may be medically unstable and require medical assessment in addition to voice prosthesis change. By having the surgeon perform both the medical assessment as well as the prosthesis change, the number of people involved in face-to-face contact is minimized. Furthermore, should the patient become unstable during the change, the surgeon is attending for medical management. Where possible, the change should happen in a negative pressure room using a PAPR if available. The room should subsequently undergo a terminal clean based on the hospital’s IPAC guidelines.

## Conclusions

Modifications to the care of the laryngectomy patient are needed during the COVID-19 pandemic. With the current technology available, most issues can be resolved temporarily without an in-person visit. Clear communication with SLP and surgeons is an important aspect of the triage process. When a face-to-face visit and change of the voice prosthesis is deemed necessary, specific protocols are required to reduce the potential transmission of the virus. Fig. 1 describes a process that can be used to begin resumption of ambulatory care service for this specialized population.

## Data Availability

Manuscript does not contain any data, and thus Not Applicable.
